# The role of omega-3 in the prevention and treatment of sarcopenia

**DOI:** 10.1007/s40520-019-01146-1

**Published:** 2019-02-19

**Authors:** Jolan Dupont, Lenore Dedeyne, Sebastiaan Dalle, Katrien Koppo, Evelien Gielen

**Affiliations:** 1 Division of Gerontology and Geriatrics, Department of Chronic Diseases, Metabolism and Ageing (CHROMETA), KU Leuven, Leuven, Belgium; 2 Exercise Physiology Research Group, Department of Movement Sciences, KU Leuven, Leuven, Belgium; 3 Department of Geriatric Medicine, UZ Leuven, Herestraat 49, 3000 Leuven, Belgium

**Keywords:** Fatty acids, Omega-3, Sarcopenia, Aged, Exercise training, Proteins

## Abstract

Sarcopenia is a geriatric syndrome with increasing importance due to the aging of the population. It is known to impose a major burden in terms of morbidity, mortality and socio-economic costs. Therefore, adequate preventive and treatment strategies are required. Progressive resistance training and protein supplementation are currently recommended for the prevention and treatment of sarcopenia. Omega-3 polyunsaturated fatty acids (PUFAs) might be an alternative therapeutic agent for sarcopenia due to their anti-inflammatory properties, which target the ‘inflammaging’, the age-related chronic low-grade inflammation which is assumed to contribute to the development of sarcopenia. In addition, omega-3 PUFAs may also have an anabolic effect on muscle through activation of the mTOR signaling and reduction of insulin resistance. This narrative review provides an overview of the current knowledge about omega-3 PUFAs and their role in the prevention and treatment of sarcopenia. We conclude that there is growing evidence for a beneficial effect of omega-3 PUFAs supplementation in sarcopenic older persons, which may add to the effect of exercise and/or protein supplementation. However, the exact dosage, frequency and use (alone or combined) in the treatment and prevention of sarcopenia still need further exploration.

## Introduction

Sarcopenia, the age-related loss of muscle mass and muscle strength, is a key feature of the aging process that predisposes elderly individuals to disability, immobility, falls, fractures and death [1]. Loss of muscle mass and strength strongly affects an older person’s independence and quality of life [2]. The term ‘sarcopenia’ was first introduced by Rosenberg in 1997 to indicate the age-related loss of muscle mass [1]. Later on, several expert groups such as the International Working Group on Sarcopenia (IWGS) and the European Working Group on Sarcopenia in Older People (EWGSOP) expanded the definition with muscle function (muscle strength or physical performance) [2, 3]. The EWGSOP defined conceptual stages of sarcopenia, i.e., ‘presarcopenia’, ‘sarcopenia’ and ‘severe sarcopenia’. To meet the criteria of sarcopenia, low muscle mass was required together with low muscle strength or low physical performance [2]. However, a consensus operational definition of sarcopenia is still lacking since the definitions of the expert groups propose different thresholds and diagnostic tests for the assessment of muscle mass, muscle strength and physical performance.

Recently, the EWGSOP revised its definition and diagnostic criteria for sarcopenia, placing muscle strength at the forefront instead of muscle mass (EWGSOP2) [4]. This new European consensus definition is in line with the growing evidence that muscle strength is better than muscle mass in predicting adverse outcomes [5]. EWGSOP2 also offers a clinically useful algorithm for case finding, diagnosis and confirmation of sarcopenia in older adults, defining new stages like ‘probable sarcopenia’, ‘confirmed sarcopenia’ and ‘severe sarcopenia’. Finally, EWGSOP2 provides clear cutoff points for the measurement of muscle mass, muscle strength and physical performance. This, together with the introduction of the ICD-10 code for sarcopenia in 2016, which was necessary to identify sarcopenia as a disease, will hopefully lead to a greater interest of clinicians to diagnose sarcopenia and of the industry to develop new therapies for sarcopenia [6–9].

Sarcopenia has an important impact on daily life activities of elderly persons. For example, due to sarcopenia almost 20% of women and almost 10% of men ≥ 65 years cannot lift a 4.5 kg weight or kneel down [10]. The condition is also found to be a significant predictor of hospitalization among older individuals [11]. Furthermore, the prevalence of sarcopenia is high, affecting up to 29% of community-dwelling adults aged ≥ 50 years, up to 10% for those in acute hospital care and between 14 and 33% of those living in long-term care institutions [12–14]. A recent longitudinal cohort study in 52 nursing homes in Belgium even suggests a higher prevalence with up to 45% of residents having sarcopenia [15]. Regardless of the applied definition, it goes without saying that sarcopenia represents an increasingly important public health concern. In 2000, the estimated healthcare cost attributable to sarcopenia in the United States was about $20 billion, corresponding to 1.5% of the total healthcare expenditures [16]. These numbers are expected to increase with the aging of the population and the concomitant rise in the prevalence of sarcopenia. A recent systematic review indeed found higher healthcare costs for patients with sarcopenia, but concluded that more research should be conducted to assess the true impact of sarcopenia on healthcare consumption [17].

Physical exercise and nutritional interventions are currently recommended for the prevention and treatment of sarcopenia [18, 19]. Progressive resistance training and protein and/or amino acids supplementation are generally accepted as the most effective approach to manage age-related muscle wasting [12, 20]. In the elderly, however, the response to physical exercise and protein intake is blunted compared to younger persons, a phenomenon referred to as anabolic resistance [21]. To counteract this anabolic resistance, guidelines recommend a higher protein intake in the elderly (≥ 65 years) as compared to the recommended daily allowance (RDA) for adults < 65 years (0.8 g protein/kg of body weight (BW)/day). Healthy older persons are recommended to consume an average daily intake of 1.0-1.2 g protein/kg BW or more [18, 22]. Older adults who suffer from acute or chronic diseases require a daily protein intake of 1.2–1.5 g/kg BW, while severely ill older adults may need up to 2.0 g protein/kg BW per day. Another strategy to stimulate muscle growth in elderly, despite the occurrence of anabolic resistance, is progressive resistance training. Finally, there is growing interest in the combined effect of physical exercise and protein supplementation on gains in muscle mass and muscle function [23, 24]. Physical activity may restore the sensitivity of older muscles to protein intake and, in turn, the ingestion of sufficient proteins in close temporal proximity to exercise produces an additional anabolic stimulus that increases the post-exercise muscle protein synthesis [25].

Another approach for sarcopenia treatment might lie in targeting inflammation. Chronic low-grade inflammation associated with aging, also referred to as ‘inflammaging’, may be an important contributor to the development of sarcopenia [26, 27]. This increased inflammatory state may form a potential therapeutic target for sarcopenia. Anti-inflammatory drugs might help to decrease inflammatory signaling and concomitantly improve physical performance in older adults [28]. Non-steroidal anti-inflammatory drugs (NSAIDs) such as piroxicam, celecoxib and ibuprofen have been tested in the past for this purpose but are currently not recommended for the treatment of sarcopenia due to the high risk on adverse events in the elderly [29–31]. Omega-3 polyunsaturated fatty acids (PUFAs) might be an alternative therapeutic agent for sarcopenia, with low risk on serious adverse events. Omega-3 PUFAs have anti-inflammatory properties and are generally known for their beneficial effects on cardiovascular risks, though a recent Cochrane review showed little or no effect of supplementation on cardiovascular health or mortality [32]. Furthermore, omega-3 PUFAs might also be beneficial for bone health, cognitive performance and eye health [33]. Finally, there is growing evidence that omega-3 PUFAs have an anabolic effect on skeletal muscle metabolism [34]. Therefore, omega-3 PUFAs supplementation may be a promising agent in the prevention and treatment of sarcopenia.

This narrative review provides an overview of the current knowledge about omega-3 PUFAs and their role in the prevention and treatment of sarcopenia. First, the age-related process of ‘inflammaging’ and the role of omega-3 PUFAs in this condition will be discussed. Next, we will clarify the mechanisms of action of omega-3 PUFAs, followed by an overview of the association between omega-3 PUFAs dietary intake, plasma levels and RBC content on the one hand and muscle mass, muscle strength and physical performance on the other hand. Finally, we will describe the results of interventional studies with omega-3 PUFAs supplementation.

## Inflammaging and its role in the development and progression of sarcopenia

Aging is often accompanied by a slight increase in plasma levels of proinflammatory mediators such as interleukin 6 (IL-6), tumor necrosis factor alpha (TNFα), c-reactive protein (CRP), interleukin 1 beta (IL-1β), and reduced levels of anti-inflammatory cytokines such as interleukin 10 (IL-10) [35]. This age-related chronic low-grade inflammation is referred to as immunosenescence or ‘inflammaging’ and involves a deteriorated immunity in elderly, making them more susceptible to infections and impeding the immune response upon infection [26].

The etiology of ‘inflammaging’ and its potential causal role in adverse health outcomes remains unclear, but most age-related diseases are associated with chronic low-grade inflammation [36]. It was suggested that increased plasma levels of proinflammatory cytokines might play a key role in the development and progression of sarcopenia, as these cytokines directly affect muscle catabolic and anabolic signaling pathways [37–40].

In their recent meta-analysis, Bano et al. concluded that sarcopenia is associated with elevated serum CRP levels, while there was no difference in IL-6 levels between sarcopenic and non-sarcopenic elderly [41]. In addition, other studies found an association between elevated CRP and sarcopenia or identified higher levels of CRP as a risk factor for the loss of muscle strength [38]. Accordingly, population-based data suggest that levels of IL-6 and TNFα are significantly elevated in Chinese sarcopenic elderly [40]. It can be concluded that data regarding inflammatory cytokines (especially IL-6) and the occurrence of sarcopenia are very limited and rather inconsistent.

IL-6 is presumed to play a dual role in the development and progression of sarcopenia. It was initially thought that this cytokine was only produced by immune cells, while later findings also identified muscle cells as IL-6 producers. In fact, muscle is a major source of circulating IL-6, especially in response to exercise [42, 43]. The role of IL-6 in the muscle remains controversial. On the one hand, when acutely increased, it might positively affect the muscle metabolism, e.g., by upregulation of the lipolysis and fat oxidation, while on the other hand it might increase the risk of sarcopenia, when chronically increased, as is the case in inflammaging.

### How to counteract this inflammaging?

The exact pathway by which inflammation causes sarcopenia is still poorly understood, but lowering inflammation with anti-inflammatory agents might offer a window of opportunities to target sarcopenia.

NSAIDs might help to decrease inflammation and improve physical performance in older adults [28]. Piroxicam is shown to improve clinically relevant measures of muscle performance and mobility (e.g., fatigue resistance) in geriatric patients hospitalized with acute infection-induced inflammation [29]. Similarly, celecoxib has a beneficial effect on fatigue resistance in these acute ill older patients [30]. In healthy untrained older adults, aged 60–85 years, 1200 mg ibuprofen a day significantly increases muscle hypertrophy in response to resistance exercise [31]. Because of the risk of adverse events (e.g., gastro-intestinal and renal toxicity and congestive heart failure) in older adults, the pros and cons of NSAIDs should be considered individually for each patient and their use is not yet recommended for this indication [30].

The effect of angiotensin converting enzyme inhibitors (ACE-inhibitors) on physical performance in the elderly has been examined, but data remain scarce and inconsistent [28]. Therefore, more research is needed to determine their role in the treatment of sarcopenia.

Omega-3 PUFAs supplementation might offer an interesting alternative opportunity, as it possesses anti-inflammatory properties and has a low risk of adverse events. Omega-3 PUFAs or n-3 PUFAs are characterized by the location of the first double bond at the third carbon. The main omega-3 PUFAs are α-linolenic acid (ALA), eicosapentaenoic acid (EPA), docosapentaenoic acid (DPA) and docosahexaenoic acid (DHA). EPA, DPA and DHA are considered long-chain PUFAs, whereas ALA is considered short-chain PUFA [44]. The concentration of EPA and DHA in cell membranes is important for ensuring structural cell function [45, 46]. ALA can be metabolized into EPA and DPA in healthy young men and women to compensate for insufficient dietary intake. This is not the case for DHA, of which sufficient intake through the diet is crucial to maintain adequate membrane DHA concentrations [45, 47]. Data from elderly persons regarding ALA metabolizing into EPA and DPA were not found.

Fatty fish (e.g., salmon and mackerel) and seafood are the main food sources for EPA and DHA. Unfortunately, these food sources are often scarce in a Western diet [44]. ALA is more abundantly present in this diet, since it is found in plant foods like nuts, chia seeds and vegetable oils (e.g., soybean oil) [44]. The Academy of Nutrition and Dietetics recommends consuming two to three servings of fatty fish a week, aiming to provide about 500 mg EPA and DHA each day. Observational research shows that most U.S. adults do not reach the recommended intake of omega-3 PUFAs, i.e., EPA and DHA. Elderly had higher intakes of EPA, but not DHA, compared to younger adults [48]. Therefore, EPA and DHA supplementation may be considered to reach the recommended daily intake.

Higher total omega-3 PUFAs plasma levels significantly reduce cardiovascular mortality, with a 35% lower risk on cardiovascular related death and an up to 69% lower risk for arrhythmic cardiovascular death [49]. Therefore, omega-3 PUFAs supplements were suggested for the prevention and treatment of cardiovascular diseases, if dietary intake is insufficient [50]. However, as mentioned before a recent Cochrane review showed inconsistent data about cardiovascular benefits of omega-3 PUFAs supplementation [32].

Omega-3 PUFAs supplementation might be beneficial for various aging processes, like cognition, bone health and eye health [33]. For example, omega-3 PUFAs supplementation reduces oxidative stress in healthy middle-aged to older U.S. adults, having a possible beneficial effect on cognition [51]. Furthermore, one study suggested that krill oil-derived omega-3 PUFAs can activate cognitive function in healthy older persons [52]. On the contrary, a larger study in 867 cognitively healthy elderly found no difference in cognitive function between elderly supplemented with either EPA + DHA or olive oil for 2 years. Thus, results are conflicting and further research is needed to confirm the effect of omega-3 PUFAs on cognition [53].

Several potential beneficial effects of omega-3 PUFAs on the muscle are suggested, like an increased nerve conduction and muscle activation, an improved mitochondrial function (energy production) or a larger myofiber size [54]. Furthermore, previous randomized controlled trials (RCTs) have shown that omega-3 PUFAs supplementation stimulates muscle protein synthesis in older adults [55, 56]. When combined with resistance training, omega-3 PUFAs supplementation augmented the increase in muscle function and quality (strength per unit muscle area) achieved by resistance training [57]. Similarly, ALA supplementation combined with resistance training resulted in a greater increase of knee flexor thickness in elderly men [58].

## Role of omega-3 in the prevention and treatment of sarcopenia

In the next paragraphs, we will describe the potential role of omega-3 in the prevention and treatment of sarcopenia. First, we will elaborate on the suggested working mechanism of omega-3 PUFAs. This will be followed by a discussion of the association between omega-3 PUFAs intake and muscle mass and function. Finally, current evidence about the effect of omega-3 PUFAs supplementation on sarcopenia components will be evaluated.

### Working mechanisms of omega-3 PUFAs

**Fig. 1:**
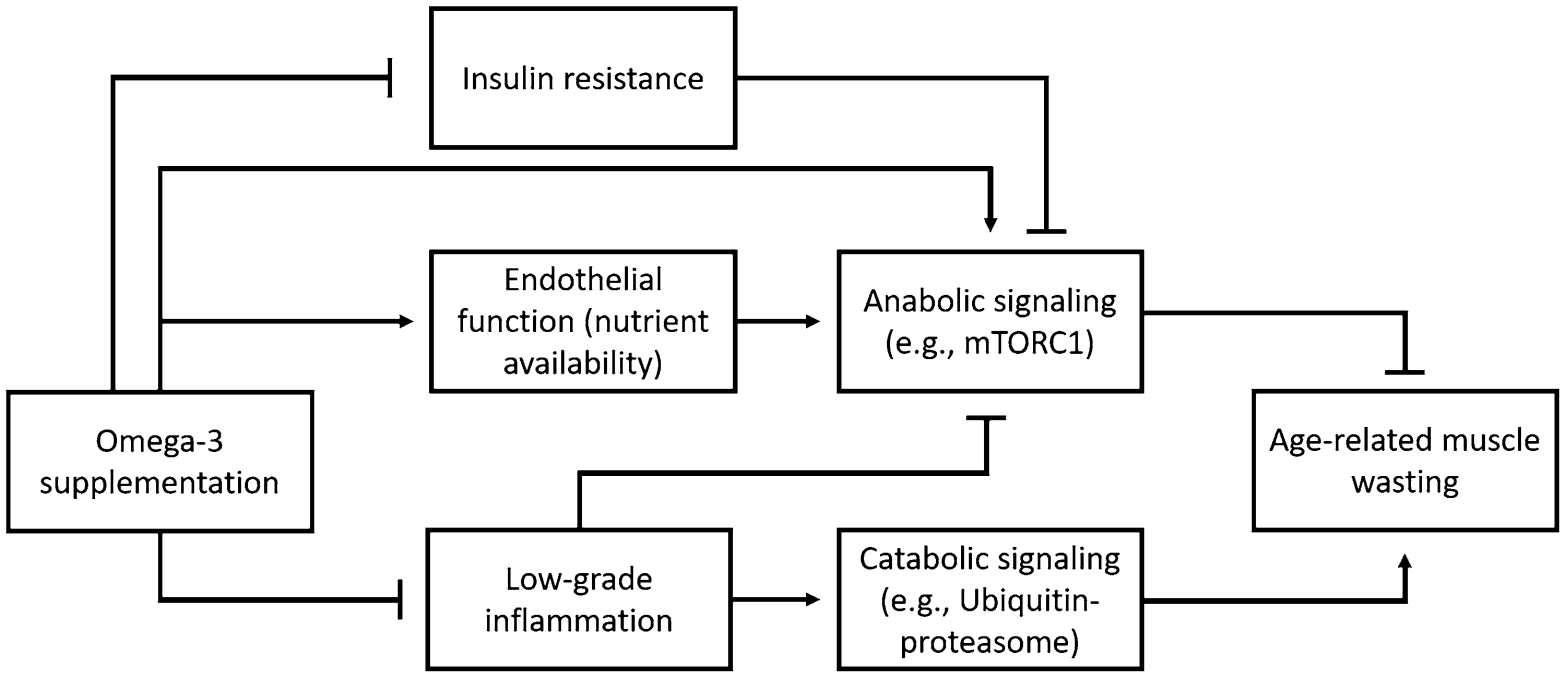
Working mechanisms of omega-3 PUFAs supplementation on sarcopenia parameters. *mTORC1* mammalian target of rapamycin complex 1

The exact mechanisms of action of omega-3 PUFAs in sarcopenia are still under research [59]. We will highlight three hypothetic mechanisms and their supporting data: anti-inflammatory effects, mammalian target of rapamycin (mTOR) activation and reduction of insulin resistance (See Fig. 1).

Anti-inflammatory effects of omega-3 PUFAs are generally accepted. In a recent meta-analysis, Custodero et al. confirmed a reduction of CRP and IL-6 after supplementation with omega-3 PUFAs in middle-aged and older adults [60]. In addition, a recent RCT investigated the effect of EPA and DHA therapy on inflammation in older adults. The supplementation had a significant lowering effect on IL-6, IL-1β and TNFα levels after 4 weeks of use and even greater after 8 weeks [61]. As previously mentioned, chronic low-grade inflammation is suggested to play a role in the development of sarcopenia. Therefore, the suppression of this low-grade inflammation is commonly presumed to be one mechanism through which omega-3 PUFAs might counteract sarcopenia. On the contrary, Smith et al. found no effect of omega-3 PUFAs supplementation on CRP, TNFα and IL-6 [55]. Further research in (pre)sarcopenic older adults is needed to confirm whether omega-3 supplementation has an effect on sarcopenia by lowering pro-inflammatory cytokines.

Another suggested working mechanism is a transcriptionally regulated anabolic effect through activation of the mTOR pathway. The mTOR pathway plays a key role in many processes of cell growth, but is mostly important for skeletal muscle generation and muscle protein synthesis. Through its downstream regulators 4E-BP1 and S6K1, mTOR regulates muscle protein synthesis [62]. Further digression in the pathways on which mTOR has an effect does not belong to the purpose of this paper, but can be reviewed in the paper of Weigl [62]. Smith et al. found an increased activation of the mTOR-p70s6k signaling pathway in response to increased amino acid and insulin supply after 8 weeks of omega-3 PUFAs compared to a placebo group [55]. Yoshino et al. found that omega-3 PUFAs supplementation induces small but important changes in the gene expression, with an increased expression of pathways involving mitochondrial function regulation and a decreased expression of inhibitory pathways on mTOR, thus in favor of skeletal muscle anabolism [56, 63]. In conclusion, omega-3 PUFAs might help to overcome the age-related anabolic resistance by increasing the rate of muscle protein synthesis through stimulation of the mTOR signaling pathway.

Finally, some research suggests a role for decreased insulin resistance in response to omega-3 PUFA therapy [54, 64]. As insulin signaling plays a key role in mTOR activation, it is conceivable that omega-3 PUFA supplementation might alleviate the anabolic resistance and thus stimulate muscle protein synthesis in the elderly population [65]. However, further research is needed to elucidate the precise mechanism(s) through which omega-3 PUFAs affect insulin sensitivity.

### Association between omega-3 PUFAs intake and muscle mass and function

Observational research suggests that a high intake of omega-3 PUFAs has a beneficial effect on physical performance in the elderly. Omega-3 PUFAs intake can be assessed through self-reported dietary intake or by measuring omega-3 PUFAs levels in plasma or red blood cell (RBC) membrane. In the next section, we will discuss the association between omega-3 PUFAs levels (assessed through self-reported dietary intake or by measuring omega-3 PUFAs plasma levels or RBC content) and sarcopenia components. The mentioned observational studies are summarized in Table 1.

**Table 1:** Overview of observational data about omega-3 PUFAs and sarcopenia

References	Year	Sample size, age (mean)	Sarcopenia defining parameter(s)	Methodology	Findings
Omega-3 PUFAs intake measured by self-reported dietary intake					
Robinson et al. [66]	2008	2893 subjects, 59–73 years (mean men 65.7 ± 2.9 years and women 66.6 ± 2.7 years)	Grip strength	Cross-sectional and retrospective cohort study	Increase in hand grip strength with 0.43 kg in men (CI 0.13–0.74) or 0.48 kg (CI 0.24–0.72) in women for each additional portion of fatty fish intake
Rousseau et al. [67]	2009	274 community-dwelling or assisted living older adults, ≥ 60 years (mean 78.9 ± 6.8 years)	Leg strength, chair rise time, handgrip strength, gait speed, timed up and go test (TUG)	Cross-sectional and retrospective cohort study	No significant effect on muscle function was found after confounder analysis
Omega-3 PUFAs intake measured by plasma levels omega-3					
Abbatecola et al. [70]	2009	884 subjects, 22–104 years (mean 68.8 ± 15.7 years)	Physical performance, measured by Short Physical Performance Battery (SPPB)	Longitudinal study with 3 years follow-up after baseline	Baseline omega3 PUFAs levels were inversely associated with the risk of developing a decline in SPPB to ≤ 9 Increased omega-6/omega-3 ratio is associated with higher risk of SPPB decline to ≤ 9
Reinders et al. [74]	2014	836 subjects (cross-sectional) 459 subjects (longitudinal), 66–96 years (mean respectively 76.7 ± 5.60 and 74.9 ± 4.98 years)	Muscle mass, knee extension strength, grip strength, muscle quality	Longitudinal study with 5 years follow-up after baseline	Cross-sectional: positive association between higher concentrations of total PUFA and larger muscle size + greater knee extension strength Longitudinal: increased ALA was related with increased knee extension strength
Reinders et al. [73]	2015	556 subjects, 66–96 years (mean 75.1 ± 5.0 years)	Gait speed	Longitudinal study with 5 years follow-up after baseline	Higher omega-3 PUFAs plasma levels, especially DHA, prevented a decrease in gait speed in women but not in men
Frison et al. [69]	2017	982 community-dwelling older adults, ≥ 65 years (mean 74.1 ± 12.4 years)	Gait speed	Cross-sectional study	Higher proportion of omega-3 PUFAs EPA and DHA are associated with a higher gait speed
Omega-3 PUFAs intake measured by RBC membrane composition					
Fougère et al. [78]	2017	1449 community-dwelling older adults with slow gait speed (< 0.8 m/s), ≥ 70 years (mean 75.2 ± 4.4 years)	SPPB	Cross-sectional study	No significant effect on muscle function was found after confounder analysis

*TUG* timed up and go test, *SPPB* Short Physical Performance Battery, *RBC* red blood cell

Two cross-sectional studies have investigated the association between self-reported dietary intake of omega-3 fatty acids and physical performance. First, Robinson et al. examined the association between food intake and grip strength in 2893 men and women aged 59–73 years included in the Hertfordshire Cohort study. They found that each additional fatty fish consumption is associated with an increase in grip strength of 0.43 kg [95% confidence interval (CI) 0.13–0.74] in men or 0.48 kg (95% CI 0.24–0.72) in women [66]. Accordingly, Rousseau et al. reported a correlation between higher omega-3 PUFAs intake (> 1.27 g daily) and increased leg strength or faster chair stand test. However, after partial correlations with protein as confounder, this association between omega-3 PUFAs intake and lower extremity function was no longer significant [67].

Omega-3 PUFAs plasma levels are often used as an indicator for dietary omega-3 intake, but results may vary due to a meal immediately prior to sample taking [68]. Four studies were found using plasma levels of omega-3 PUFAs as a parameter of dietary intake. First, Frison et al. showed that among 982 older adults, those with a higher omega-3 PUFAs plasma level are less likely to have a lower gait speed (< 0.63 m/s) [69]. Secondly, Abbatecola et al. showed in a longitudinal study that lower baseline omega-3 PUFAs levels increase the risk of a decline in Short Physical Performance Battery (SPPB) score to ≤9, compatible with low physical performance. Increased omega-3 PUFAs plasma levels seemed to protect against accelerated decline of physical performance, whereas a higher omega-6/omega-3 ratio was associated with increased risk of developing poor physical performance and slower gait speed [70]. This omega-6/omega-3 ratio is often used to assess cardiovascular risk, though the beneficial or harmful role of omega-6 PUFA remains uncertain [71, 72]. Third, a longitudinal study of Reinders et al. suggested that higher omega-3 PUFAs plasma levels, especially DHA, prevented a significant decrease in gait speed in women but not in men [73]. More research is needed to further elucidate whether omega-3 PUFAs might exert sex-specific effects. Another study of Reinders et al. described a cross-sectional association between higher concentrations of total PUFA and larger muscle size and greater knee extension strength. Remarkably, greater ALA levels were associated with decreased intermuscular adipose tissue, whereas EPA levels increased intermuscular adipose tissue [74]. Intermuscular adipose tissue is often used as a parameter for muscle quality and is independently associated with lower muscle strength [75, 76]. The previously mentioned studies of Frison et al. and Abbatecola et al. did not investigate the amount of intermuscular adipose tissue, so further research is required to determine the association between omega-3 PUFAs plasma levels and intermuscular adipose tissue [69, 70].

As a parameter for omega-3 PUFAs intake researchers also often use the omega-3 index or red blood cell (RBC) membrane content of omega-3 PUFAs (mostly EPA and DHA) [77]. This can be used to measure the compliance with the intake of omega-3 PUFAs supplements [78, 79]. Only one study investigated the association between the omega-3 index and physical performance. Fougère et al. found that a low omega-3 index was associated with lower physical performance, measured by the SPPB in older adults with a mean age of 75 years. However, after confounder analysis for age, gender, grip strength, body mass index and geriatric depression scale, results were non-significant, indicating that the beneficial effect of omega-3 PUFAs on physical performance might be influenced by confounders [78].

Thus, observational data show that a higher omega-3 PUFAs intake or higher plasma levels/RBC content of omega-3 PUFAs are associated with higher muscle mass, muscle strength, muscle quality and physical performance, all of which are important determinants for sarcopenia. In future research about sarcopenia, a systematic evaluation of omega-3 plasma or RBC levels might be useful as an indicator of omega-3 dietary intake.

### Effects of omega-3 PUFAs supplementation on muscle mass and muscle function

To investigate the potential therapeutic role of omega-3 PUFAs supplementation in sarcopenic elderly, an increasing number of clinical trials have been and are being performed with omega-3 PUFAs supplements, either alone either in combination with an exercise intervention. This paper will discuss available RCTs and their results. We will start with RCTs investigating omega-3 PUFAs supplementation alone, followed by RCTs investigating a combination with exercise and finally discuss some data of the triple combination of omega-3 PUFAs supplementation, protein supplementation and exercise. An overview of these RCTs can be found in Table 2.

**Table 2:** Overview of interventional data about omega-3 PUFAs supplementation and sarcopenia

References	Year	Sample size, age (years)	Sarcopenia defining parameter(s)	Methodology	Findings
Omega-3 PUFAs supplementation alone					
Hutchins-Wiese [80]	2011	126 postmenopausal women, 64–96 years (mean 75 ± 6 years)	Gait speed, hand grip strength, chair rise time	RCT, 1.2 g EPA + DHA vs olive oil placebo daily for 6 months	Increase in walking speed with 0.03–0.05 m/s in omega-3 PUFAs group No significant benefit for hand grip strength of chair rise time
Smith et al. [55]	2011	16 healthy older adults, ≥ 65 years (mean 71 ± 2 years)	Muscle protein synthesis	RCT, 1.86 g EPA + 1.50 g DHA vs corn oil placebo daily for 8 weeks	Omega-3 PUFAs supplementation increased the muscle protein synthesis in response to a hyperaminoacidemic-hyperinsulinemic clamp
Smith et al. [56]	2015	60 healthy older adults, 60–85 years (mean control group 69 ± 7 year; omega-3 group 68 ± 5 years)	Thigh muscle volume, hand grip strength, one-repetition maximum (1-RM) strength	RCT, 1.86 g EPA + 1.50 g DHA vs corn oil placebo daily for 6 months	Omega-3 PUFAs supplementation increases thigh muscle volume and muscle strength
Krzyminska-Siemaszko et al. [83]	2015	735 community-dwelling older adults, ≥ 65 years (mean 74.6 ± 8 years)	Muscle mass, gait speed, TUG	RCT, 660 mg EPA + 440 mg DHA + 200 mg other omega-3 PUFAs + 10 mg vitamin E vs 11 mg vitamin E alone daily for 12 weeks	No significant effect on muscle mass, muscle strength or gait speed
Omega-3 PUFAs supplementation combined with physical exercise					
Cornish and Chilibeck [58]	2009	51 subjects, > 60 years (mean 65.4 ± 0.8 years)	Muscle thickness (knee and elbow), muscle strength (1-RM)	RCT, 14 g/day ALA vs corn oil daily combined with resistance training program (3days/week) for 12 weeks	ALA supplementation gave a larger increase in knee flexor muscle thickness
Rodacki et al. [84]	2012	45 women (mean 64 ± 1.0 years)	Muscle strength (knee flexor/extensor, plantar extensors, dorsiflexors), chair rise time, TUG	RCT, fish oil containing 0.4 g EPA + 0.3 g DHA daily as supplementation, 3 arms: strength training (ST) alone for 90 days, ST 90 days + 90 days supplementation vs ST 90 days + 150 days supplementation	All groups increased strength due to the strength training, but the association of a supplement caused greater improvements in muscle strength and functional capacity There was no extra increase due to an additional 60 days of supplementation (90 vs 150 days)
Da Boit et al. [57]	2012	50 subjects, ≥ 65 years (mean men 70.6 ± 4.5 years; women 70.7 ± 3.3 years)	Muscle strength (knee extensor), SPPB, muscle quality (strength per unit muscle area)	RCT, 2.1 g EPA + 0.6 g DHA vs safflower oil placebo daily, combined with resistance training 2 ×/w for 18 weeks	Omega-3 PUFAs supplementation augments increases in muscle function and quality in older women but not in older men after resistance exercise training
Omega-3 PUFAs supplementation combined with protein supplementation and physical exercise					
Zhu et al. [86]	2018	113 community-dwelling sarcopenic elderly, ≥ 65 years (mean control 72.2 ± 6.6 years; exercise 74.5 ± 7.1 years; combined group 74.8 ± 6.9 years)	Gait speed, chair rise time, hand grip strength, muscle strength (leg extensors), muscle mass (lower limb, appendicular skeletal mass)	RCT, 0.29 g omega-3 PUFA + 8.61 g protein + 1.21 g β-hydroxy β-methyl butyrate, 130 IU vitamin D twice daily, 3 arms: exercise alone for 12 weeks; combined exercise and supplement for 12 weeks; control	No significant effect on gait speed was found Chair rise time and muscle strength improved in both interventions groups Nutritional supplementation added benefit in increasing lower limb muscle mass and appendicular skeletal mass after 12 weeks

*RCT* randomized control trial, *1-RM* one-repetition maximum, *ST* strength training, *SPPB* Short Physical Performance Battery, *TUG* timed up and go test

Four RCTs investigated the effect of omega-3 PUFAs supplementation alone in older adults. First, Hutchins-Wiese et al. found that 6 months of fish oil-derived supplementation (containing 1.2 g EPA and DHA a day) resulted in a significant increase in walking speed with 0.03–0.05 m/s in postmenopausal women [80]. This small change is considered to be clinically relevant in the context of aging [81, 82]. Secondly, Smith et al. found that 8 weeks of omega-3 PUFAs supplementation (containing 1.86 g EPA and 1.5 g DHA a day) in healthy older adults (≥ 65 years) increased the muscle protein synthesis in response to a hyperaminoacidemic-hyperinsulinemic clamp to a larger extend than compared to a corn oil supplemented group [55]. This observation suggests that omega-3 PUFAs supplementation improved the sensitivity to an anabolic stimulus (i.e., amino acids and insulin) and decreased the age-related anabolic resistance. The same research group demonstrated that 6 months omega-3 PUFAs supplementation (again containing 1.86 g EPA and 1.5 g DHA a day) significantly increased thigh muscle volume and muscle strength compared to a placebo in healthy older adults (60–85 years) [56]. Finally, Krzymińska-Siemaszko et al. could not demonstrate an effect of 12 weeks of omega-3 PUFAs supplementation (containing 660 mg EPA and 440 mg DHA a day) on muscle mass, muscle strength or gait speed in Polish older adults suffering from a decreased muscle mass [83]. The authors attribute this to the low sample size of their study and the lower dosage of EPA and DHA compared to the dosages used in the aforementioned studies of Hutchins-Wiese et al. and Smith et al. [55, 56, 80]. Additionally, duration of supplementation and characteristics of the study population might also contribute to the conflicting results.

Overall, the effect of omega-3 PUFAs supplements alone seems promising for sarcopenia by enhancing muscle protein synthesis, improving gait speed and increasing muscle strength and physical performance.

Furthermore, increasing evidence suggests that omega-3 PUFAs supplementation amplifies the effect of physical exercise on parameters of muscle mass, strength and performance. Three studies investigated the combined effect of omega-3 supplementation and an exercise intervention. In 2009, Cornish and Chilibeck combined 12 weeks of ALA supplementation with a resistance training program (three times a week) in older adults (mean age 65.4 ± 0.8 years) [58]. Muscle thickness of the elbow flexor, elbow extensor and knee extensor as well as chess and leg press strength significantly increased following resistance training, but no differences were observed between the ALA and the placebo group. The only benefit of additional ALA supplementation was a significantly larger increase in knee flexor muscle thickness. Secondly, Rodacki et al. randomly assigned healthy older women (aged 64 ± 1.4 years) to three groups: 90 days of strength training only, strength training combined with 90 days of fish oil supplementation or strength training combined with 150 days of supplementation (starting 60 days before intervention) [84]. The different duration of supplementation was to investigate whether the duration of supplementation modifies the effect. Strength training increased muscle strength in all groups, however, when combined with omega-3 PUFA supplements the effect on muscle strength and functional capacity was enhanced. There was no significant difference in effect between 90 days or 150 days of omega-3 PUFAs supplementation. Third, Da Boit et al. examined the effect of a resistance training program with omega-3 PUFAs supplements [57]. Older men and women (aged ≥ 65 years) received supplements or placebo for 18 weeks, whilst performing a lower-limb resistance training twice weekly. Omega-3 PUFAs supplementation significantly augmented the increase in muscle function and quality (strength per unit muscle area) achieved by resistance training in older women, but not in men.

Combining omega-3 supplements with an exercise intervention might be a promising therapeutic agent for sarcopenia, with omega-3 supplements enhancing the effects of the exercise intervention on muscle mass and muscle function.

Up to now, only two studies have examined the combined effect of omega-3 PUFAs, exercise and protein supplements, though one of them in patients with chronic obstructive pulmonary disease (COPD) [85]. However, the supplement did not only consist of omega-3 PUFAs, so the specific omega 3-PUFA effect remains unclear. Recently Zhu et al. published a study in Chinese sarcopenic elderly, combining a training program (twice a week in-group, once a week at home) and a nutrition supplement twice daily for 12 weeks [86]. The supplement contained 8.61 g protein, 1.21 g β-hydroxy β-methyl butyrate, 130 IU vitamin D and 0.29 g omega-3 PUFAs. When the nutritional supplement was combined with the exercise intervention, the authors found an additional effect on appendicular skeletal mass and lower limb muscle mass, compared to the exercise intervention alone. However, the authors acknowledge the very low dosage of omega-3 PUFAs. Therefore, it is unclear whether omega-3 PUFAs did contribute to the positive effects. In sarcopenic older persons, there are currently no other data available about the effect of the combination of protein supplementation, exercise and omega-3 PUFAs supplementation.

## Conclusion

Sarcopenia is a geriatric syndrome with increasing importance due to the aging of the population. It is known to impose a major burden in terms of morbidity, mortality and socio-economic costs. Therefore, adequate preventive and treatment strategies are required. Progressive resistance training and protein supplementation are currently recommended for the prevention and treatment of sarcopenia.

Inflammaging may play an important role in the development of sarcopenia. This might offer a new target for the prevention and treatment of sarcopenia. Omega-3 PUFAs, which lower inflammation but which also may enhance mTOR signaling and reduce insulin resistance, are positively associated with muscle mass, muscle strength, muscle quality and physical performance. Therefore, omega-3 PUFAs supplements may be a potential therapy or preventive measure for sarcopenia, either alone or combined with the classic therapeutic strategies. The effect of omega-3 PUFAs supplements alone seems promising for sarcopenia by improving muscle mass, muscle strength and physical performance. In combination with an exercise intervention, omega-3 PUFAs supplementation might augment the increase in muscle mass and function obtained by the exercise intervention. The triple combination of exercise, protein and omega-3 PUFAs supplementation needs more research, especially in persons with sarcopenia, but omega-3 PUFAs might be a promising enhancer of the effects of physical exercise and protein supplementation. More research is needed to confirm these findings.

We conclude that there is growing evidence for a beneficial effect of omega-3 PUFAs supplementation in sarcopenic older persons. However, the exact dosage, frequency and use (alone or combined) in the treatment and prevention of sarcopenia still need further exploration.
